# Streptococcus salivarius endophthalmitis and corneal ring infiltrate after intravitreal bevacizumab injection

**DOI:** 10.3205/oc000270

**Published:** 2026-05-05

**Authors:** Kemal Örnek, Tülay Karacan Erşekerci

**Affiliations:** 1 Department of Ophthalmology, School of Medicine, Kırşehir Ahi Evran University, Kırşehir, Turkey

**Keywords:** endophthalmitis, corneal ring infiltrate, Streptococcus salivarius, intravitreal bevacizumab

## Abstract

A 65-year-old male patient received intravitreal bevacizumab for age-related macular degeneration. On the first day, he presented with ocular pain, corneal stromal infiltration and dense vitritis. Prompt intravitreal vancomycin and ceftazidime were injected after sampling. Widespread gram-positive cocci were seen on microscopy after staining the vitreous sample. No significant improvement took place the following day and the cornea was completely opacified. The patient underwent an early vitrectomy combined with penetrating keratoplasty. Culture grew *Streptococcus salivarius*. Postoperative visual acuity was hand motions and the retina was attached.

## Introduction

Bevacizumab is a humanized recombinant monoclonal antibody developed against VEGF-A. It is used intravitreally in the treatment of various retinal diseases. Sight-threatening complications of intravitreal injection include uveitis, retinal tear, rhegmatogenous retinal detachment, traumatic cataract, intraocular hemorrhage, and endophthalmitis. Endophthalmitis has the worst visual prognosis among these. The causative pathogen is mostly bacteria (90%). Eighty percentage of the isolated microorganisms are gram-positive, and the majority are coagulase-negative staphylococci [1].

Here, we present the first case of rapid onset *Streptococcus salivarius* endophthalmitis and corneal ring infiltrate after intravitreal bevacizumab injection.

## Case description

A 65-year-old male patient applied to receive his ongoing intravitreal treatment for age-related macular degeneration. Best corrected visual acuity was 7/10 in the right eye and 1/10 in the left eye. He had 1+ nuclear sclerosis in both eyes. There was choroidal neovascularization on the left. Optical coherence tomography imaging revealed subretinal fluid with foveal thickening. Local anesthesia was provided with 0.5% proparacaine drops. The eyelids, eyelashes, and ocular surface were sterilized with povidone-iodine. Then, 0.5 mg/ml intravitreal bevacizumab was injected 4 mm behind the limbus temporally. Topical moxifloxacin drop was initiated after the procedure. 

**Figure 1:**
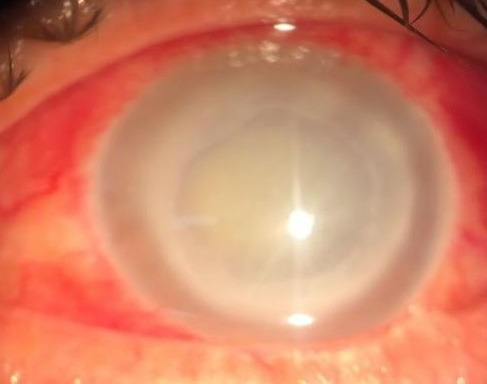
Slit-lamp examination of the eye demonstrating diffuse conjunctival injection, corneal ring infiltrate and vitreous haze

**Figure 2:**
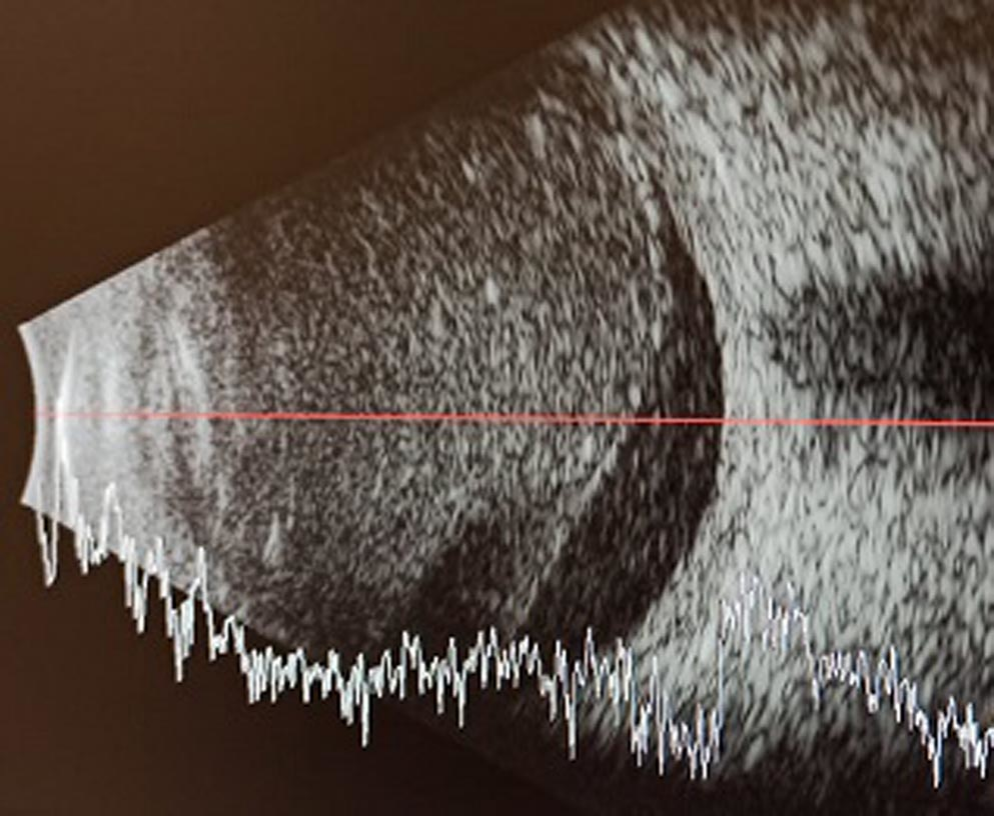
B-scan ultrasonography showing dense opacity throughout the vitreous cavity with attached retina

The patient presented with decreased vision and ocular pain on the first day. Visual acuity was hand motions. Slit-lamp examination showed lid swelling, diffuse conjunctival injection, corneal ring infiltrate, anterior chamber cells, vitreous inflammation, and blunted red reflex. There was no hypopyon (Figure 1 (Fig. 1)). B-Scan ultrasonography revealed dense opacity throughout the vitreous cavity, suggestive of vitritis with attached retina (Figure 2 (Fig. 2)). The patient was afebrile without any systemic symptoms. He denied any ocular trauma, but had a tooth extracted the day before the injection and was under dental implant treatment. He did not use any systemic antibiotics. 

Given the worsening of the vision and presenting clinical findings, immediate vitreous tap was performed and vitreous sample was obtained. Intravitreal 1 mg/0.1 ml vancomycin and 2 mg/0.1 ml ceftazidime were injected after vitreous tap. Medical treatment was planned as hourly fortified ceftazidime and vancomycin eye drops, cyclopentolate 3 times a day and oral ciprofloxacin 750 mg tablets twice a day.

Microscopic examination and gram staining of the vitreous sample revealed widespread gram-positive cocci. The culture grew a large number of *Streptococcus salivarius*. The antibiogram showed that the causative pathogen was sensitive to the previously administered intravitreal antibiotics. No significant improvement was observed in the patient’s symptoms and findings, and the keratitis spread to the central tissue and the cornea became completely opaque. Therefore, the patient underwent an early pars plana vitrectomy combined with penetrating keratoplasty. Corneal sample confirmed the *Streptococcus salivarius* infection. Postoperative visual acuity was hand movements, and the retina was attached. The patient is under close follow-up with intraocular silicone tamponade and corneal graft without any further complications.

## Discussion

Infectious endophthalmitis is an uncommon complication of intravitreal injection, but when it occurs, the visual outcome can be devastating. Some microorganisms, particularly streptococcus species, tend to be more virulent than others [2], [3]. There are very few reports that implicate *Streptococcus salivarius* directly in cases of endophthalmitis [4], [5].

Streptococcus species cause severe endophthalmitis leading to profound vision loss [2], [3]. Kurniawan et al. published the results of patients with streptococcal endophthalmitis, 35.6% of whom applied with hand motions and 42.6% with light perception. Visual acuity was below 20/200 in 77.6% of patients, and 24.7% of eyes received evisceration [2]. In another series by Kuriyan et al, 75% of eyes had visual acuity below 20/400 and 25% had evisceration [3]. 

Hyperacute endophthalmitis is a severe, rapidly progressing intraocular infection, often presenting within 24 hours, causing severe vision loss and potential loss of the eye. The most common cause is severe infection by gram-negative bacteria (especially *Pseudomonas aeruginosa*) or *Streptococcus pneumoniae*. Due to the rapid destruction of intraocular tissues, the prognosis is generally poor and can sometimes lead to blindness or removal of the eye (evisceration). 

In a recent study, all cases of intravitreal injection-related endophthalmitis presented with ocular pain, decreased visual acuity and vitritis on average 3.4 days after injection. Only 18 of 23 cases (78%) had a hypopyon [6]. In this case, there was no hypopyon at the initial examination. The rapid onset of corneal infiltrate and intraocular inflammation, beginning on day 1 after intravitreal injection and causing decreased visual acuity, raised significant clinical concerns. However, there were no history of uveitis and clinical findings of toxic anterior segment syndrome such as increased intraocular pressure, diffuse corneal edema and lack of isolated organisms. 

*Streptococcus salivarius* is classified as a member of the *viridans streptococci* group. It is a commensal bacterium that predominates in the oral cavity of healthy individuals. In this case, the patient had a tooth extraction and dental treatment the day before the injection. Systemic investigations revealed no evidence of infectious pathogens prior to the dental procedures. However, the vitreous culture of the patient grew a large number of *Streptococcus salivarius*. We assume that the patient may be at risk for *Streptococcus salivarius* that could have spread from the oral cavity via the bloodstream and contaminated the injection site. Another route of spread could be through conversations between the patient and staff before the injection, which could have led to eye contamination. Therefore, possible spread of bacteria from the neighbouring tissues, high bacterial load which determines disease severity along with virulence factors and host immune response might have caused the very early onset of ocular inflammation in this case. 

Corneal ring infiltrate is a ring-shaped stromal infiltrate, circumferential to the limbus, typically leaving a clear zone from it. Ring infiltrates are commonly associated with keratitis caused by Acanthamoeba and fungal keratitis. They are usually present after 24–48 hours of inoculation. Corneal ring infiltrates related to endophthalmitis were described with several infectious agents (*Pseudomonas aeruginosa*, *Staphylococcus aureus*), but the most severe cases are associated with *Bacillus cereus* [7], [8]. Ring infiltration secondary to polymicrobial infection, caused by *Streptococcus salivarius*, *Streptococcus oralis*, and a coagulase-negative *Staphylococcus* sp., has also been reported after postoperative contact lens use [9]. Here, we present a rare case of corneal ring infiltrate associated with *Streptococcus salivarius* endophthalmitis following intravitreal bevacizumab injection. 

## Conclusion

This is the first reported case of rapid onset *Streptococcus salivarius* endophthalmitis with corneal ring infiltrate after intravitreal bevacizumab injection. Early diagnosis and prompt treatment are critical to prevent devastating consequences. Treatment of post-injection endophthalmitis should be tailored to the individual case depending on the clinical presentation. In such cases, early surgical intervention seems to be preferable. In addition to this, patients who are planned to have intravitreal injections should be informed to postpone dental treatments unless classified as emergency. 

## Notes

### Informed consent

This report was conducted in accordance with the Declaration of Helsinki. Informed consent, including permission for publication of the images included, was provided before the injection.

### Competing interests

The authors declare that they have no competing interests.
